# Thermo-Responsive Fluorescent Polymers with Diverse LCSTs for Ratiometric Temperature Sensing through FRET

**DOI:** 10.3390/polym10030283

**Published:** 2018-03-08

**Authors:** Zhaoyang Ding, Chunfei Wang, Gang Feng, Xuanjun Zhang

**Affiliations:** Faculty of Health Sciences, University of Macau, Macau SAR 999078, China; zhaoyangding@umac.mo (Z.D.); yb67596@umac.mo (C.W.); yb67608@umac.mo (G.F.)

**Keywords:** *N*-isopropylacrylamide, *N*-isopropylmethacrylamide, ratiometric temperature sensing, FRET

## Abstract

Temperature is a significant parameter to regulate biological reactions and functions inside cells. Sensing the intracellular temperature with a competent method is necessary to understand life science. In this work, an energy-transfer polymeric thermometer was designed for temperature sensing. The thermometer was prepared from two thermo-responsive polymers with different lower critical solution temperatures (LCSTs) of 31.1 °C and 48.6 °C, coupling with blue and red fluorescent molecules, respectively, developed for ratiometric temperature sensing based on the Förster resonance energy transfer (FRET) mechanism. The polymers were synthesized from two monomers, *N*-isopropylacrylamide (NIPA) and *N*-isopropylmethacrylamide (NIPmA), which provided different temperature responses. The fluorescent intensity of each polymer (peaked at 436 and 628 nm, respectively) decreased upon the heating of the polymer aqueous solution. While these two polymer aqueous solutions were mixed, the fluorescent intensity decrease at 436 nm and substantial fluorescence enhancement at 628 nm was observed with the increasing temperature due to FRET effect. The cell imaging of HeLa cells by these thermo-responsive polymers was explored. The difference of LCSTs resulting in ratiometric fluorescence change would have a potential impact on the various biomedical applications.

## 1. Introduction

Temperature is an essential physiological parameter during all biochemical reactions in living cells. It has a close relationship with cellular functions, such as cell division, gene expression, metabolism, enzyme reaction, and so on [1,2]. In addition, various abnormal medical phenomena, like cancer cell growth, are often accompanied by a temperature increase. Owing to these considerable demands, lots of promising approaches to fluorescent thermometers for cell temperature sensing have been studied [3]. Recently, many works have been developed for this requirement, including small organic dyes [4], quantum dots [5,6], polymers [7,8,9,10,11], and gold nanoclusters [12].

Nowadays, stimuli-responsive polymers have received more attentions for their potential biomedical applications. A variety of stimuli-responsive polymers whose properties change in response to temperature, pH, ionic strength, light, and chemical stress have been investigated [13,14,15,16,17]. Thermo-responsive polymers have been applied in the fields of sensors, catalyst supports, drug carriers, and bio-separation [18,19,20]. Thermo-responsive polymers based on *N*-isopropylacrylamide (NIPA) were the most common used in these years. These polymers have a lower critical solution temperature (LCST) close to human and most animal’s physiological temperature, with good biocompatibility and low toxicity [21]. Many fluorophores [11,22,23] have already been introduced into NIPA-based polymers to visualize the temperature in living cells as fluorescent thermometer. 

**Scheme 1:**
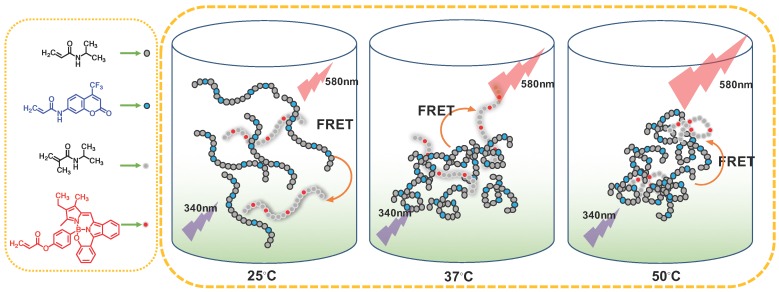
Fabrication of two thermo-responsive fluorescent polymers with diverse LCSTs.

Fluorescent temperature sensing is usually based on the fluorescence intensity changes at certain wavelengths instead of fluorescence lifetime owing to its accessibility and prevalence. However, the fluorescence intensity at a single wavelength can be easily affected. Ratiometric fluorescent temperature sensing is more and more popular with potential advantages. For the ratiometric fluorescent temperature sensing based on the fluorescence resonance energy transfer (FRET) mechanism, multi-color fluorescence of NIPA-based polymer temperature sensors were reported [24,25,26,27]. In these studies, only one thermo-responsive monomer NIPA was used, which might limit the range of the temperature sensor. When a methyl group is added to NIPA, such as *N*-isopropylmethacrylamide (NIPmA), the LCST of the NIPmA-based polymer was higher at around 45 °C to 50°C [28,29]. The introduction of NIPmA to the NIPA-based temperature sensing system is expected to enlarge the range of the temperature sensing, which should undergo a reversible LCST phase transition between the temperature range from 25 °C to 50°C. With the increasing of temperature, the shrink speed of the NIPA-based polymer was faster than the NIPmA-based polymer, leading to the distance change and FRET change between two polymers as shown in Scheme 1. 

To test this hypothesis, we designed a NIPA-based polymer with blue fluorescence molecule (7-[4-(Trifluoromethyl)coumarin]methacrylamide) and a NIPmA-based polymer with red fluorescence molecule (BOBPYBX) synthesized by ourselves to form a novel polymeric ratiometric temperature sensor through FRET by the mixing of these two polymer solutions. We also characterized the effects of the temperature on their fluorescent behavior and exhibiting good performance both in aqueous solution and in the temperature imaging of cells.

## 2. Materials and Methods

### 2.1. Materials

*N*-isopropylacrylamide (NIPA), *N*-isopropylmethacrylamide (NIPmA), 7-[4-(trifluoromethyl)coumarin]methacrylamide (TCMA), 2,2′-azobis(isobutyronitrile) (AIBN), 3-mercaptopropionic acid (MPA), 2-hydroxyphenyboronic acid, tetrakis(triphenylphosphine)palladium (Pd(PPh_3_)_4_), Phosphorus(V) oxybromide (POBr_3_) and phosphorus(V) oxychloride (POCl_3_) were purchased from Sigma-Aldrich (St. Louis, MO, USA). 1-Isoindolinone, 2,4-dimethyl-3-ethylpyrrole, 4-hydroxyphenylboronic acid, acryloyl chloride, and triethylamine were purchased from Dieckman (Hong Kong) Chemical Industry Company Ltd. (Hong Kong, China). All other reagents and solvents were of analytical grade. 

### 2.2. Synthetic of BOBPYBX

**Scheme 2:**
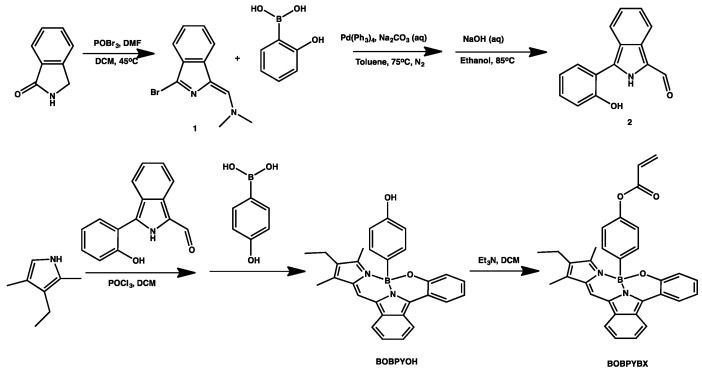
Synthetic procedure of BOBPYBX.

**1** as presented in Scheme 2 was synthesized according to previous literatures with modification [30,31]. A solution of POBr_3_ (20 mmol, 5.73 g) in anhydrous dichloromethane (5 mL) was added dropwise to Dimethylformamide (DMF) (20 mmol, 1.46 g) in anhydrous dichloromethane (15 mL) at 0 °C. The mixture was stirred for 30 min at room temperature. Then a solution of 1-isoindolinone (10 mmol, 1.33 g) in anhydrous dichloromethane (50 mL) was added to the mixture at 0 °C. Subsequently, the reaction mixture was heated at reflux for 6 h. After cooling, the solvent was removed at reduced pressure. Ice water was added, and aqueous NaOH (5 M) was added the mixture to a pH of around 8. A black solid precipitated and the mixture was stirred overnight, then **1** (yield was 84.2%) was collected by filtration.

**2** as presented in Scheme 2 and BOBPYOH were synthesized by modifying previous method [32]. To **1** (8.00 mmol, 2 g), to 2-hydroxyphenyboronic acid (17.50 mmol, 2.41 g) and Pd(PPh_3_)_4_ (0.25 mmol, 0.29 g) aqueous Na_2_CO_3_ (1 M, 20 mL) and dry toluene (50 mL) were added in a Schlenk flask under nitrogen. This reaction mixture was then degassed via three freeze-pump-thaw cycles before filling with nitrogen again. The Schlenk flask was heated to 75 °C for 24 h. After cooling to room temperature, the reaction mixture was washed with water (30 mL × 3). Organic layers were combined, dried over anhydrous Na_2_SO_4_, and evaporated under vacuum. The crude product was refluxed for 3 h in ethanol (120 mL) containing aqueous NaOH (4 M, 20 mL). The solvent was removed in vacuum. The resultant solid was dissolved in ethyl acetate (100 mL), and neutralized with HCl (3 M). Organic layers were combined, dried under anhydrous Na_2_SO_4_, filtrated, and concentrated under vacuum, and the residual product was purified by silica gel column chromatography (dichloromethane/ethyl acetate = 1:1) to afford **2** (yield was 75.8%). POCl_3_ (5.2 mmol, 0.8 g) was added to a dichloromethane solution (15 mL) of 2,4-dimethyl-3-ethylpyrrole (10.4 mmol, 1.28 g) at 0 °C. Then a solution of **2** (5.2 mmol, 1.23 g) in dichloromethane (25 mL) was added dropwise to the reaction mixture at 0 °C. The reaction mixture was stirred at room temperature for 4 h. To this solution, 4-hydroxyphenylboronic acid (52 mmol, 7.17 g) was dissolved in THF and added. Then the reaction mixture was stirred for another 4 h. After evaporation of the solvent, the residual product was purified by silica gel column chromatography (dichloromethane/*n*-hexane = 10:1) to give BOBPYOH (yield was 72.3%). NMR results of BOBPYOH were shown in Figures S1 and S2. ^1^H NMR (400 MHz, CDCl_3_) δ = 8.12 (d, *J* = 8.2, 1H), 7.97 (dd, *J* = 7.9, 1.5, 1H), 7.88 (d, *J* = 8.1, 1H), 7.47 (t, *J* = 7.4, 1H), 7.44–7.38 (m, 2H), 7.34 (d, *J* = 7.2, 2H), 7.04 (d, *J* = 8.4, 2H), 7.00–6.94 (m, 1H), 6.54–6.47 (m, 2H), 2.46 (s, 3H), 2.39 (dd, *J* = 7.6, 1.6, 2H), 2.27 (s, 3H), 1.04 (t, *J* = 7.6, 3H). ^13^C NMR (101 MHz, CDCl_3_) δ = 157.11, 154.20, 150.96, 143.18, 135.34, 133.23, 133.00, 132.90, 132.10, 130.36, 128.77, 128.43, 126.35, 125.65, 125.38, 123.43, 120.13, 119.65, 119.57, 119.08, 115.92, 114.10, 67.99, 25.62, 17.50, 13.07, 9.59.

BOBPYBX was synthesized by modifying hydroxyl group with acryloyl chloride [33]. To a solution of BOBPYOH (2 mmol, 0.86 g) and triethylamine (3 mmol, 0.3 g) in dichloromethane (20 mL) acryloyl chloride (20 mmol, 1.8 g) was added slowly and stirred for 0.5 h at 0 °C. Then the reaction was stirred further at room temperature for 12 h. The mixture was treated with water and the organic layer was separated. The aqueous layer was extracted with dichloromethane and the combined organic layer was washed with saturated aqueous NH_4_Cl, brine, and dried over anhydrous Na_2_SO_4_. The solvent was removed by rotary evaporation and the residue was purified by silica gel column chromatography (dichloromethane/*n*-hexane = 10:1) to afford BOBPYBX (yield was 35.5%). NMR results of BOBPYBX were shown in Figures S3 and S4. ^1^H NMR (400 MHz, CDCl_3_) δ = 8.12 (d, *J* = 8.2, 1H), 7.97 (dd, *J* = 7.9, 1.5, 1H), 7.89 (d, *J* = 8.1, 1H), 7.48 (t, *J* = 7.5, 1H), 7.44–7.38 (m, 2H), 7.34 (dd, *J* = 11.2, 4.0, 1H), 7.28 (d, *J* = 0.9, 1H), 7.20 (d, *J* = 8.4, 2H), 7.02–6.93 (m, 1H), 6.83–6.73 (m, 2H), 6.48 (dd, *J* = 17.3, 1.4, 1H), 6.22 (dd, *J* = 17.3, 10.4, 1H), 5.90 (dd, *J* = 10.4, 1.4, 1H), 2.47 (s, 3H), 2.39 (tt, *J* = 9.2, 4.5, 2H), 2.27 (s, 3H), 1.05 (t, *J* = 7.6, 3H). ^13^C NMR (101 MHz, CDCl_3_) δ = 164.68, 156.91, 151.01, 149.31, 143.29, 135.33, 133.43, 133.09, 132.66, 132.03, 131.94, 130.46, 128.74, 128.55, 128.24, 126.39, 125.67, 125.47, 123.47, 120.14, 119.86, 119.81, 119.60, 119.07, 115.97, 17.48, 14.84, 13.05, 9.59.

### 2.3. Synthesis of Fluorescent Polymers

Two thermo-responsive polymers were named from the monomers, P_NB_ consisted of NIPA and TCMA, and P_NmR_ was NIPmA and BOBPBYX. P_NB_ was prepared using radical polymerization, as shown in Scheme 2. NIPA (1.58 g, 14 mmol) and TCMA (4.3 mg, 0.015 mmol) were dissolved in ethanol (20 mL). AIBN (10 mg, 0.06 mmol) and MPA (50 mg, 0.5 mmol), which act as the radical initiator and the chain transfer agent to control the molecular weight of the polymers, respectively, were added to the solution. The reaction mixture was degassed and reacted in a N_2_ atmosphere at 65 °C for 12 h. After the reaction finished, the solvent ethanol was removed by a vacuum dryer and the product was precipitated by *n*-hexane and washed with acetone three times. The solid product was dried under vacuum conditions. The molecular weight of the polymer was determined by gel permeation chromatography (GPC) analysis. P_NmR_ was prepared from NIPmA and BOBPYBX, which was synthesized by ourselves as shown in Scheme 2, according to the above procedure.

### 2.4. Analytical Techniques

GPC was conducted on a Cirrus system (PL-GPC 50, Santa Clara, CA, USA) equipped with a differential refractive index detector. DMF was used as the eluent with a gel column (300 × 650 mm^2^) (at 40 °C, the flow rate = 1 mL/min). A Fourier transform infrared spectrometer (FTIR, IRAffinity-1S, Shimadzu, Japan) was used to determinate the structures of the fluorescent polymers. For each sample, each spectrum was obtained by 32 scans with the wavenumber ranging from 400 to 4000 cm^−1^ and resolution was 4 cm^−1^.

LCSTs of the polymers were determined by measuring the optical transmittance of their aqueous solution (0.5% *w*/*v*). A sample cell with a 1 cm path length was used to measure the transmittance using 500 nm over a range of temperatures from 25 to 50 °C using a UV−Vis spectrophotometer (Shimadzu UV-3600 spectrophotometer, Shimadzu, Japan). The temperature at which the transmittance decreased to half of its initial value was taken as the LCST [34]. Fluorescence spectra (Fluorolg-4 spectrofluorometric) with a temperature controller were performed for the experiments. A quartz cuvette with a 1 cm path length was used. The maximum excitation wavelengths of the fluorescent polymers were as follows (0.5% *w*/*v* aqueous solution): P_NB_ λex = 340 nm; P_NmR_ λex = 580 nm; P_MIX_ λex = 340 nm. The effects temperature on the fluorescence intensity of the polymers were evaluated between 25 and 50 °C. 

### 2.5. Cell Culture

The cervical cancer line HeLa cells were kindly provided by Faculty of Health Sciences, University of Macau. HeLa cells were incubated in DMED (Dubecco’s Modified Eagle Medium) medium supplemented with 10% FBS (fetal bovine serum) and 1% penicillin-streptomycin solution. All cells were maintained at 37 °C, in a 5% CO_2_ humidified environment. 

HeLa cells were seeded into glass-bottomed culture dishes and allowed to adhere for 24 h. After removing the medium, 10 μM polymer solution was added and incubated for 1 h at 37 °C. After the incubation, cells were washed twice by pre-warmed PBS buffer. Fixative solution (Histochoice^®^ Mb Tissue Fixative, Amresco, Dallas, TX, USA) was added for 15 min at room temperature, then washed three with PBS buffer. The samples were observed by Carl Zeiss Confocal LSM710 at 25 °C or 37 °C.

## 3. Results and Discussion

**Scheme 3:**
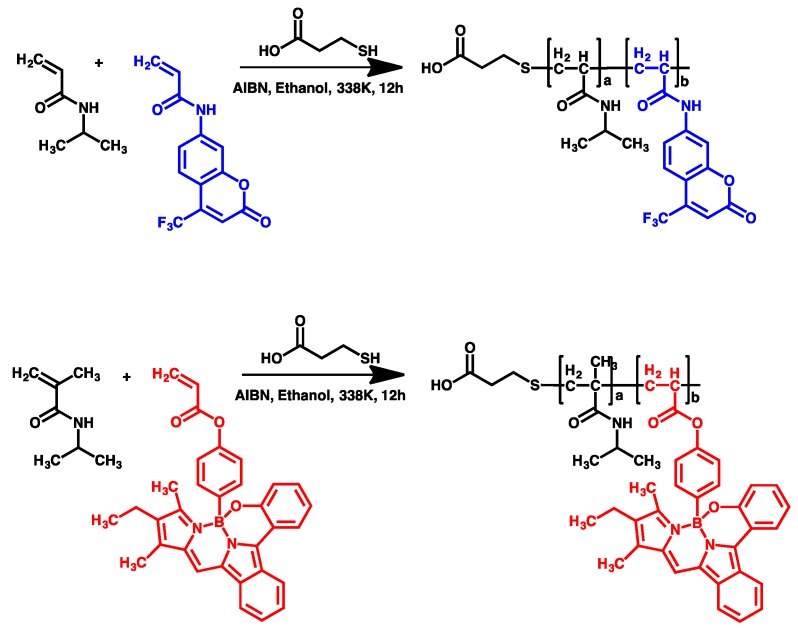
Syntheses of fluorescent polymers.

Two fluorescent polymers were synthesized via radical polymerization. The synthetic scheme and structures of the polymers are shown in Scheme 3. For these two polymers, NIPA and NIPmA, were selected as the backbone of the thermo-responsive fluorescent polymers, to ensure the polymers having enough thermo-responsibility and water-solubility, the NIPA or NIPmA units in polymers were controlled above 98 mol % and less than 1 mol % of the fluorescent monomers were introduced into the polymers. 7-[4-(Trifluoromethyl)coumarin]methacrylamide (blue) and newly-synthesized BOBPYBX (red) were chosen as fluorescent monomers to form the FRET system. The polymers undergo reversible LCSTs phase transition between the temperature range from 25 °C to 50 °C, which covers the physiological temperature of humans and most animals. 

**Figure 1:**
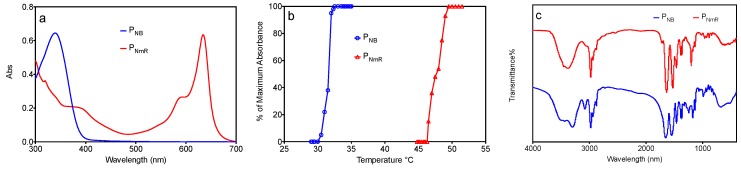
Characterization of fluorescent polymers. (**a**) Absorption spectra of the fluorescent polymers; (**b**) temperature-dependent for the fluorescent polymers in aqueous solution (500 nm, 0.5% *w*/*v*); and (**c**) FT-IR results of the fluorescent polymers.

The successful synthesis of fluorescent polymers was confirmed by FT-IR and absorption measurement as shown in Figure 1. According to the results, the maximum absorption peaks of P_NB_ and P_NmR_ were 340 nm and 620 nm, respectively. The LCSTs of P_NB_ and P_NmR_ were 31.1 °C and 48.6 °C, which were as expected. The FTIR spectrum of P_NB_ featured the characteristic absorption peaks at 3300–3090 cm^−1^ (secondary amine group), 2980 cm^−1^ (methyl group), 2900 cm^−1^ (methylene group), and 1180–1130 cm^−1^ (trifluoromethyl group), which are attributable to the two monomers. This strongly indicates the successful formation of polymer P_NB_. The FT-IR spectrum of P_NmR_ also exhibited the similar result. The fluorescent monomer ratio was so small that did not show very typical peaks in the FT-IR results, however, the absorption spectrum and fluorescent spectrum proved the polymerization was successful. The results of number-averaged and weight-averaged molecular weights were determined by gel permeation chromatography (GPC), as shown in Table 1 and Figures S5 and S6. The oligomeric products were obtained due to the use of chain-transfer agent.

**Figure 2:**
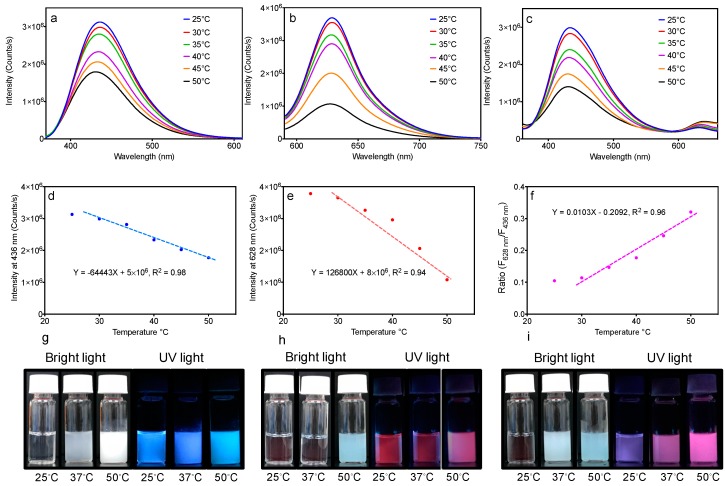
Fluorescence responses of polymers to temperature from 25 °C to 50 °C. (**a**) Fluorescence spectra of P_NB_ upon temperature 25 °C to 50 °C; (**b**) fluorescence spectra of P_NmR_ upon temperature 25 °C to 50 °C; (**c**) fluorescence spectra of P_MIX_ upon temperature 25 °C to 50 °C; (**d**) Linear correlation of P_NB_ between the emission intensity at 436 nm and temperature; (**e**) linear correlation of P_NmR_ between the emission intensity at 628 nm and temperature; (**f**) linear correlation of P_MIX_ between the emission intensity (F628 nm/F436 nm) and temperature; (**g**–**i**) naked eye photographs of the fluorescence change of the polymers P_NB_, P_NmR_, and P_MIX_ in aqueous solution (0.5% *w*/*v*) at different temperatures, respectively.

We then investigated the temperature response behavior of the fluorescent polymers. The temperature-dependent fluorescence spectra and optical transmittance of P_NB_ in aqueous solution are shown in Figure 2a. The fluorescent intensity of P_NB_ was found to decrease with increasing of the temperature from 25 °C to 50 °C, and the polymer P_NmR_ showed the similar phenomenon as in Figure 2b. As shown in Figure 2d,e, the fluorescent intensity of both P_NB_ and P_NmR_ had the liner relationship with the temperature from 30 °C to 50 °C. Hydrate form and swollen state of the thermo-responsive polymers were driven by their native properties. The fluorescent monomer displayed higher emission quantum yield in polar media, resulting in the stronger fluorescence intensity with the increase of the temperature. We could, therefore, conclude that the fluorescent intensity responses of these polymers were driven by a combination of the properties of both thermo-responsive monomers and the fluorescent monomers. The LCSTs of P_NB_ and P_NmR_ were 31.1 and 48.6 °C, respectively. Due to these results, ratiometric fluorescent temperature sensing through FRET which is very powerful in analysis and sensing can be achieved by mixing these two polymers, especially between their two LCSTs. We tried several ratios of mixed P_NB_ and P_NmR_, and the final ratio of P_NB_ and P_NmR_ was set as 20:1. The fluorescence response results were indicated in Figure 2c, a dramatic fluorescence decrease at 436 nm and substantial fluorescence enhancement at 628 nm was observed upon heating. However, the fluorescence enhancement of P_NmR_ was limited in comparison with the result in Figure 2b. The ratio of fluorescent intensity F628 nm/F436 nm had liner relationship with temperature between two LCSTs as shown in Figure 2f. With the increasing of temperature, the shrink speed of P_NB_ was faster than P_NmR_, leading to the distance change and FRET change between two polymers. The shrink changes and fluorescence changes of the polymers to different temperatures could be directly visualized by the naked eye, and the fluorescence was excited by the light of hand-held UV lamp at 365 nm. For P_NB_ shown in Figure 2g, the polymer solution became turbid from 25 °C to 37 °C and 50 °C, and the fluorescence were all blue emissions. In Figure 2h, P_NmR_ solution was clear at 25 °C and 37 °C, turbid when at 50 °C which was above the LCST of it and the P_NmR_ solution exhibited dark red emission. P_MIX_ exhibited purple emission at 25 °C, pink and bright pink emission at 37 °C and 50 °C due to FRET effects, as shown in Figure 2i. In the meantime, the P_MIX_ solution became turbid from 25 °C to 37 °C and 50 °C.

**Figure 3:**
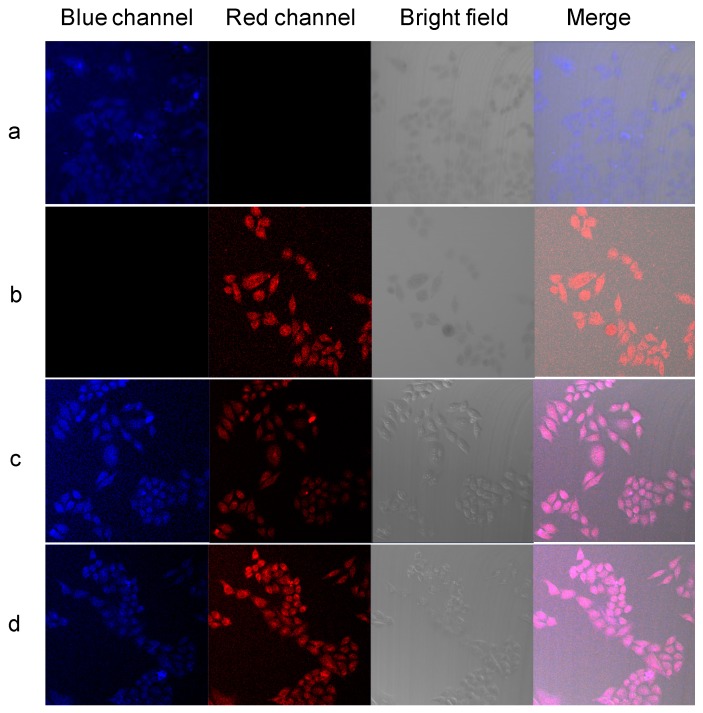
Fluorescent images of HeLa cells incubated with different polymers: (**a**) incubated with P_NB_; (**b**) incubated with P_NmR_; and (**c**,**d**) incubated with P_MIX_; (**a**–**c**) were observed at 25 °C and (**d**) was observed at 37 °C.

In view of the significance of temperature on the biological events within living cells, fluorescent images of HeLa cells incubated with polymers were explored. Figure 3a showed blue emission of cells based on the uptake of P_NB_ and Figure 3b showed a red emission of cells because of the uptake of P_NmR_. When the cells were incubated with P_MIX_, both blue and red emissions were observed as shown in Figure 3c,d, and the merge picture showed pink emission. Figure 3c had stronger blue emission and weaker red emission compared with Figure 3d due to the change of observing temperature from 25 °C to 37 °C. The results were consistent with previous fluorescent spectrum data. 

## 4. Conclusions

In summary, two fluorescent polymers, P_NB_ and P_NmR_, were prepared to form a novel energy-transfer thermometer for ratiometric temperature sensing. They exhibit different LCSTs because of the various thermo-responsive monomers. The temperature sensing system displayed good stability and biocompatibility. The intracellular temperature imaging of living cells by these ratiometric temperature sensing polymers was explored. It is expected that this LCSTs difference showing ratiometric fluorescence change would have a significant impact on the various applications, such as fluorescent temperature sensing and bio-imaging of biological processes at specific organelles. 

## Figures and Tables

**Table 1:** GPC results of fluorescent polymers.

Polymers	*M*_n_	*M*_w_	PDI
P_NB_	5957	7954	1.33121
P_NmR_	11,144	16,140	1.44831

*M*_w_: weight average molecular weight; *M*_n_: number average molecular weight; PDI: Polymer dispersity index, PDI = *M*_w_/*M*_n_.

## Supplementary Materials

The following are available online at http://www.mdpi.com/2073-4360/10/3/283/s1, Figure S1: ^1^H NMR of BOBPYOH, Figure S2: ^13^C NMR of BOBPYOH, Figure S3: ^1^H NMR of BOBPYOX, Figure S4: ^13^C NMR of BOBPYOX. Figure S5: GPC result of P_NB_, Figure S6: GPC result of P_NmR_.
