# Supplementary material for: Thermo-Responsive Fluorescent Polymers with Diverse LCSTs for Ratiometric Temperature Sensing through FRET
Source: Polymers (Basel). 2018 Mar 8;10(3):283. doi: 10.3390/polym10030283 (PMC6415166; doi:10.3390/polym10030283)
Supplement: Supplementary file 1 [file polymers-10-00283-s001.pdf]

Supporting Information

Thermo-responsive fluorescent polymers with diverse LCSTs for ratiometric temperature sensing through FRET

Zhaoyang Ding[§], Chunfei Wang[§], Gang Feng, Xuanjun Zhang[*]

Faculty of Health Sciences, University of Macau, Macau SAR, China

*Corresponding author. xuanjunzhang@umac.mo

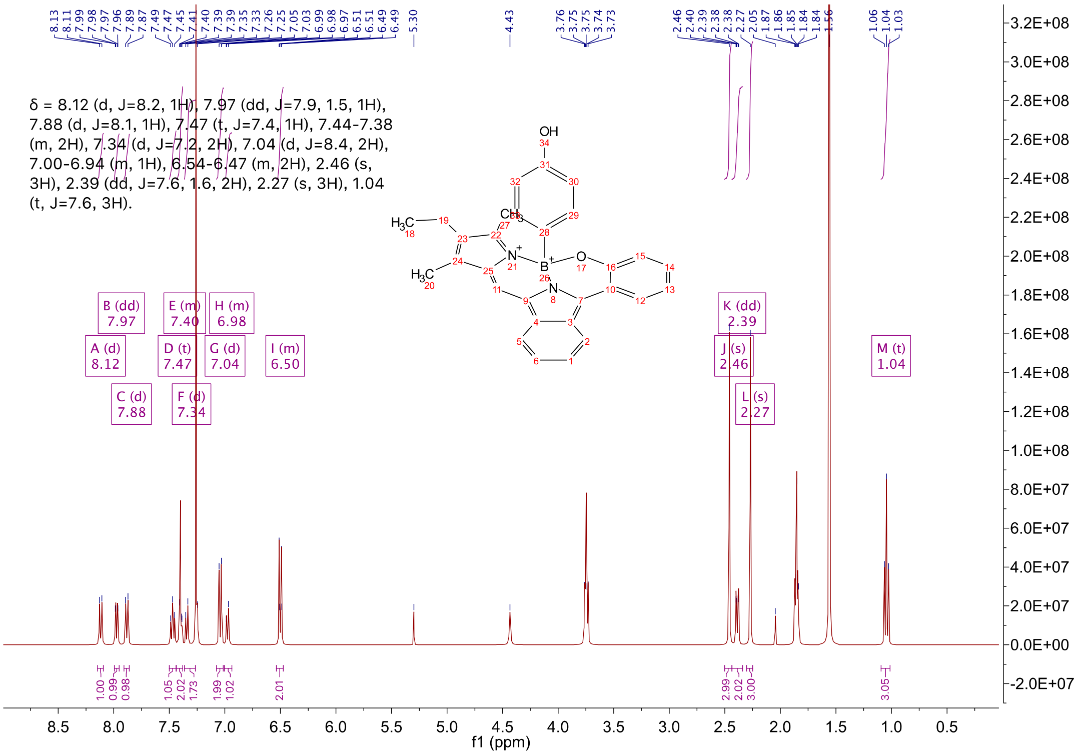

Figure S1. ^{1}H **NMR of BOBPYOH**

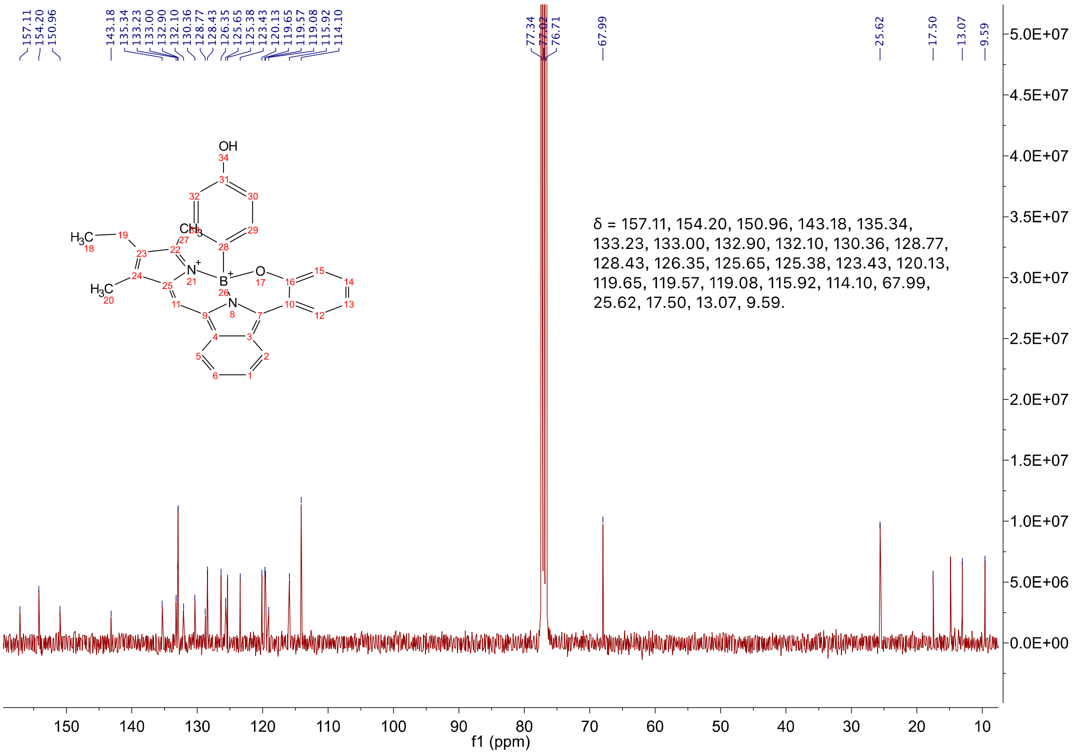

Figure S2. ^{13}C **NMR of BOBPYOH**

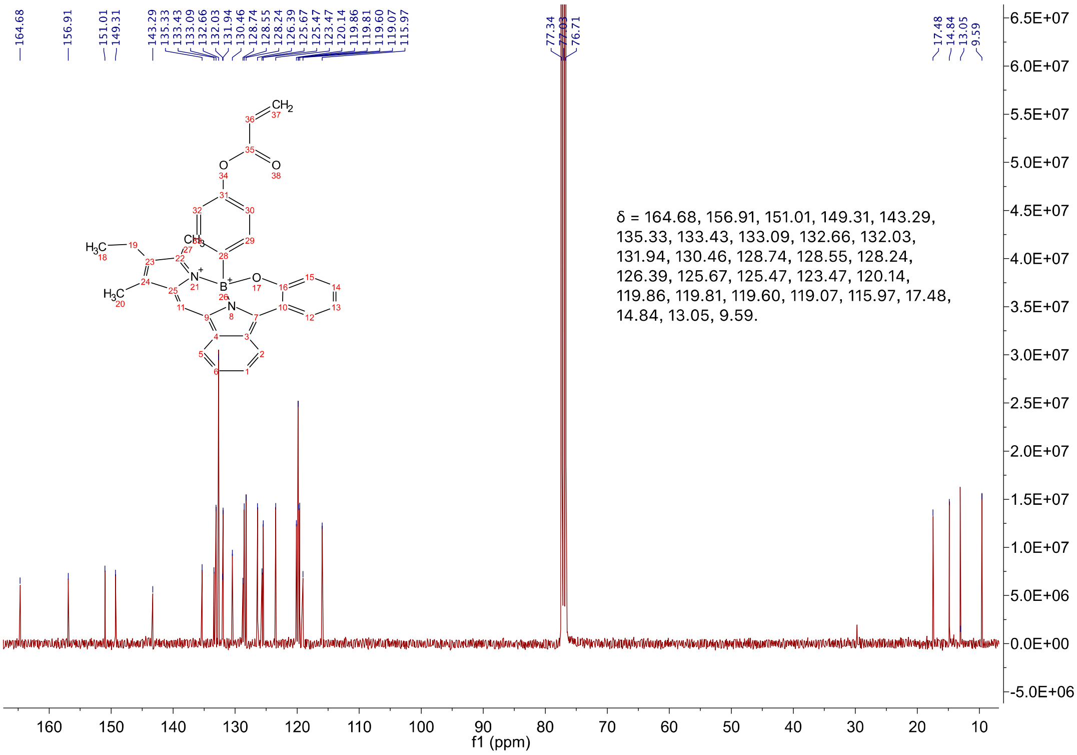

Figure S3. ^{1}H NMR of BOBPYOX

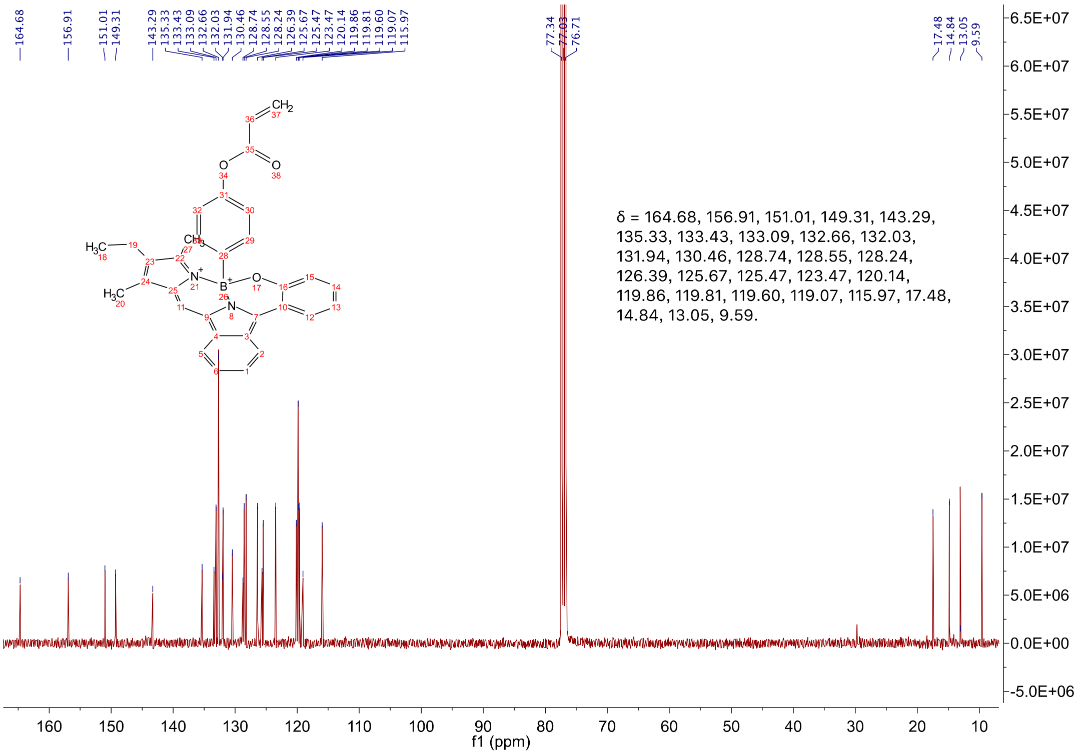

Figure S4. ^{13}C NMR of BOBPYOX

Workbook Details

Eluent: DMF

Injection Volume: 100.0 ul

Detector: RI

Flow Rate: 1.00 ml/min

Column Set Length: 650 mm

Temperature: 50

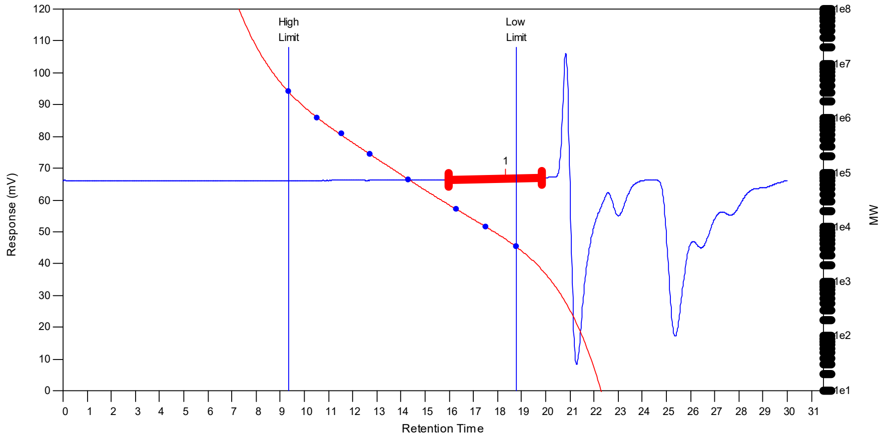

MW Averages

Peak No	Mp	Mn	Mw	Mz	Mz+1	Mv	PD
1	8015	5975	7954	10107	12175	7646	1.33121

Processed Peaks

Peak No	Name	Start RT (mins)	Max RT (mins)	End RT (mins)	Pk Height (mV)	% Height	Area (mV.secs)	% Area
1		15.97	18.35	19.85	0.802255	0	107.656	100

Figure S5. GPC result of P_{NB}

Workbook Details

Eluent: DMF

Injection Volume: 100.0 ul

Detector: RI

Flow Rate: 1.00 ml/min

Column Set Length: 650 mm

Temperature: 50

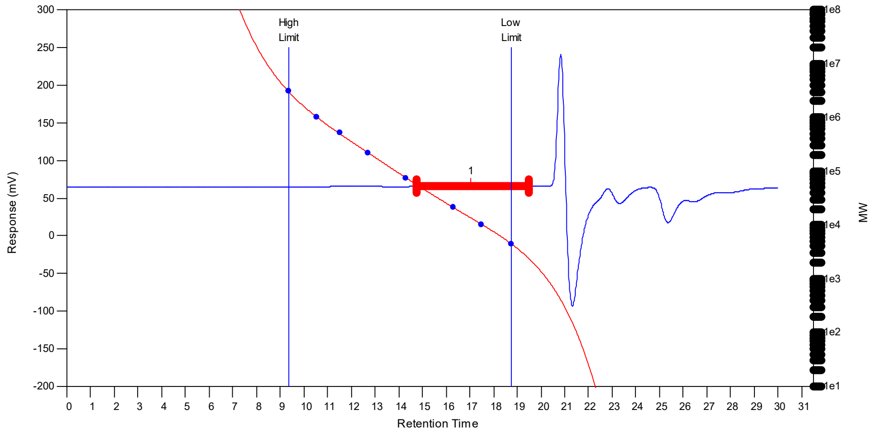

MW Averages

Peak No	Mp	Mn	Mw	Mz	Mz+1	Mv	PD
1	14626	11144	16140	21913	27399	15334	1.44831

Processed Peaks

Peak No	Name	Start RT (mins)	Max RT (mins)	End RT (mins)	Pk Height (mV)	% Height	Area (mV.secs)	% Area
1		14.73	17.02	19.50	1.54918	0	255.827	100

Figure S6. GPC result of P_{NmR}
